# On Generalization Based on Bi et al. Iterative Methods with Eighth-Order Convergence for Solving Nonlinear Equations

**DOI:** 10.1155/2014/272949

**Published:** 2014-01-19

**Authors:** Taher Lotfi, Alicia Cordero, Juan R. Torregrosa, Morteza Amir Abadi, Maryam Mohammadi Zadeh

**Affiliations:** ^1^Department of Applied Mathematics, Hamedan Branch, Islamic Azad University, Hamedan 65188, Iran; ^2^Instituto de Matemática Multidisciplinar, Universitat Politècnica de València, Camino de Vera, s/n, 46022 Valencia, Spain

## Abstract

The primary goal of this work is to provide a general optimal three-step class of iterative methods based on the schemes designed by Bi et al. (2009). Accordingly, it requires four functional evaluations per iteration with eighth-order convergence. Consequently, it satisfies Kung and Traub's conjecture relevant to construction optimal methods without memory. Moreover, some concrete methods of this class are shown and implemented numerically, showing their applicability and efficiency.

## 1. Introduction

Multipoint methods for solving nonlinear equations *f*(*x*) = 0, where *f* : *D* ⊂ *R* → *R*, possess an important advantage since they overcome theoretical limits of one-point methods concerning the convergence order and computational efficiency [1–5].

During the last years, there have been many attempts to construct optimal three-step iterative methods without memory for solving nonlinear equations. Indeed, Bi et al. [6, 7] are pioneers in this case, after Kung and Traub [8]. Some other optimal methods are due to Cordero et al. [9–11], Dzunic et al. [12, 13], Heydari et al. [14], Geum and Kim [15–17], Kou et al. [18], Liu and Wang [19–21], Sharma and Sharma [22], Soleimani et al. [4], Soleymani [23], Soleymani et al. [24–27], Thukral [28–30], and Thukral and Petković [31]. Recently, iterative methods for root finding have been used for finding matrix inversion arising from linear systems; for more details consult Wang [32], Babajee et al. [33], Montazeri et al. [34], Soleymani [35, 36], Thukral [37], and the references therein.

In this paper we present a new optimal class of three-step methods without memory, which employs the idea of weight functions in the second and third steps. The order of this class is eight requiring four functional evaluations per step and therefore it supports Kung and Traub's conjecture [8]. The proposed class includes the Bi et al. methods [6, 7].

In order to design the new methods, we will use the divided differences. Let *f*(*x*) be a function defined on an interval *I*, where *I* is the smallest interval containing *k* + 1 distinct nodes *x*_1_, *x*_2_,…, *x*_*k*_. The divided difference *f*[*x*_0_, *x*_1_,…, *x*_*k*_] with *k*th-order is defined as follows: *f*[*x*_0_] = *f*(*x*_0_),

(1) f[x0]=f[x1]−f[x0]x1−x0,…,f[x0,x1,…,xk]=f[x1,x2,…,xk]−f[x0,x1,…xk−1]xk−x0.

It is clear that the divided difference *f*[*x*_0_, *x*_1_,…*x*_*k*_] is a symmetric function of its arguments *x*_0_, *x*_1_,…, *x*_*k*_. Moreover, if we assume that *f* ∈ *C* ^(*k*+1)^(*I*_*x*_), where *I*_*x*_ is the smallest interval containing the nodes *x*_0_, *x*_1_,…*x*_*k*_, and *x*, then *f*[*x*_0_, *x*_1_,…, *x*_*k*_, *x*] = *f* ^(*k*+1)^(*ξ*)/(*k* + 1)! for a suitable *ξ* ∈ *I*_*x*_. Specially, if *x*_0_ = *x*_1_ = ⋯ = *x*_*k*_ = *x*, then

(2) $$\begin{array}{c} f\left[x,x,\ldots ,x,x\right]=\frac{f^{\left(k+1\right)}\left(x\right)}{\left(k+1\right)!}. \end{array}$$

Moreover, we recall the so-called efficiency index defined by Ostrowski [38] as EI = *p* ^1/*n*^, where *p* is the order of convergence and *n* is the total number of functional evaluations per iteration.

## 2. Main Result: Development and Convergence Analysis of the New Methods

It is well known that Newton's method converges quadratically under standard conditions. To obtain a higher order of convergence and higher efficiency index than that of Newton's scheme, we compose Newton's method twice as follows:

(3) yn=xn−f(xn)f′(xn),  zn=yn−f(yn)f′(yn),xn+1=zn−f(zn)f′(zn), n=0,1,2,….

As this scheme is eighth-order convergent but its efficiency is poor, we need to reduce the number of functional evaluations. In the third step, *f*′(*z*_*n*_) can be approximated in a similar way as in [6].

Consider

(4) $$\begin{array}{c} f^{\prime }\left(z_{n}\right)\approx f\left[z_{n},y_{n}\right]+f\left[z_{n},x_{n},x_{n}\right]\left(z_{n}-y_{n}\right). \end{array}$$

Also, a “frozen” derivative can be used in the second step and adequate weight functions will improve the efficiency in the second and last steps. So, the following three-step methods are proposed:

(5) $$\begin{array}{c} y_{n}=x_{n}-\frac{f\left(x_{n}\right)}{f^{\prime }\left(x_{n}\right)},\;\;z_{n}=y_{n}-g\left(s_{n}\right)\frac{f\left(y_{n}\right)}{f^{\prime }\left(x_{n}\right)},\\ s_{n}=\frac{f\left(y_{n}\right)}{f\left(x_{n}\right)},\\ x_{n+1}\;=z_{n}-h\left(t_{n}\right)\frac{f\left(z_{n}\right)}{f\left[z_{n},y_{n}\right]+f\left[z_{n},x_{n},x_{n}\right]\left(z_{n}-y_{n}\right)},\\ t_{n}=\frac{f\left(z_{n}\right)}{f\left(x_{n}\right)}. \end{array}$$

It is clear that the proposed methods by (5) require only four functional evaluations per iteration, while they are not eighth-order methods, in general. To recover the optimal eighth-order, we find some suitable conditions on the introduced weight functions *g*(*s*_*n*_) and *h*(*t*_*n*_).

To find the weight functions *g* and *h* in (5) providing order eight, we will use the method of undetermined coefficients and Taylor's series about 0, since *t*_*n*_ → 0, *s*_*n*_ → 0, when *n* → *∞*.

Let us consider

(6) $$\begin{array}{c} g\left(s_{n}\right)\approx g\left(0\right)+g^{\prime }\left(0\right)s_{n}+g^{\prime \prime }\left(0\right)\frac{s_{n}^{2}}{2},\\ h\left(t_{n}\right)\approx h\left(0\right)+h^{\prime }\left(0\right)t_{n}. \end{array}$$

The following result states suitable conditions for proving that the new class has eighth-order of convergence.

**Theorem 1**

Assume that *f* is a sufficiently differentiable real function. Let one suppose that *α* ∈ *D* is a simple zero of *f*. If the initial estimation *x*_0_ is close enough to *α*, then the sequence {*x*_*n*_} generated by any method of the family (5) converges to *α* with eighth-order of convergence if *g* and *h* are real sufficiently differentiable functions satisfying *g*(0) = *h*(0) = 1, *g*′(0) = *h*′(0) = 2, and *g*′′(0) = 10.

**Proof**

Let us introduce the following notations:

(7) en=xn−α,  eyn=yn−α,  ezn=zn−α,en+1=xn+1−α,  ci=1i!f(i)(α)f′(α), i≥2.

Using Taylor's expansion and taking into account *f*(*α*) = 0, we have

(8) f(xn)=f′(α)[en+c2en2+c3en3+c4en4+c5en5+c6en6+c7en7+c8en8]+O(en9).

Also by direct differentiation, we obtain

(9) f′(xn) =f′(α)[1+2c2en+3c3en2+4c4en3+5c5en4+6c6en5+7c7en6+8c8en7]+O(en8).

From (8) and (9) we get

(10) f(xn)f′(xn) =en−c2en2+2(c22−c3)en3+(7c2c3−4c23−3c4)en4  +(8c24−20c22c3+6c32+10c2c4−4c5)en5  +[−16c25+52c23c3−28c22c4+17c3c4    +c2(−33c32+13c5)−5c6]en6  +2[16c26−64c24c3−9c33    +36c23c4+6c42+9c22(7c32−2c5)    +11c3c5+c2(−46c3c4+8c6)−3c7]en7  +[−64c27+304c25c3−176c24c4    −75c32c4+31c4c5+c23(−408c32+92c5)    +4c22(87c3c4−11c6)+27c3c6    +c2(135c33−64c42−118c3c5+19c7)−7c8]  ×en8+O(en9).

Hence,

(11) eyn=c2en2+2(−c22+c3)en3+(−7c2c3+4c23+3c4)en4 −(8c24−20c22c3+6c32+10c2c4−4c5)en5   −[−16c25+52c23c3−28c22c4     +17c3c4+c2(−33c32+13c5)−5c6]en6   −2[16c26−64c24c3−9c33+36c23c4+6c42     +9c22(7c32−2c5)+11c3c5     +c2(−46c3c4+8c6)−3c7]en7   −[−64c27+304c25c3−176c24c4     −75c32c4+31c4c5+c23(−408c32+92c5)     +4c22(87c3c4−11c6)+27c3c6     +c2(135c33−64c42−118c3c5+19c7)−7c8] ×en8+O(en9).

Similar to (8),

(12) f(yn) =f′(α)[c2en2+2(−c22+c3)en3+(5c23−7c2c3+3c4)en4       −2(6c24−12c22c3+3c32+5c2c4−2c5)en5       +(28c25−73c23c3+34c22c4−17c3c4         +c2(37c32−13c5)+5c6)]en6+O(en7).

Moreover, taking into account (8), (9), and (12),

(13) sn=f(yn)f(xn) =c2en+(−3c22+2c3)en2+(8c23−10c2c3+3c4)en3  +(−20c24+37c22c3−8c32−14c2c4)en4  +(48c25−118c23c3+55c2c32+51c22c4−22c3c4)en5  ×(−112c26+344c24c3−252c22c32+26c33   −163c23c4+150c2c3c4−15c42)en6+O(en7),

(14) f(yn)f′(xn)=c2en2+(−4c22+2c3)en3    +(13c23−14c2c3+3c4)en4    +(−38c24+64c22c3−20c2c4+4(−3c32+c5))    ×en5+O(en6).

By using Taylor's expansion around zero

(15) $$\begin{array}{c} g\left(s_{n}\right)\approx g\left(0\right)+g^{\prime }\left(0\right)s_{n}+\frac{g^{\prime \prime }\left(0\right)}{2}s_{n}^{2}, \end{array}$$

and by using (11)–(15),

(16) $$\begin{array}{c} e_{z_{n}}=A_{2}e_{n}^{2}+A_{3}e_{n}^{3}+A_{4}e_{n}^{4}+O\left(e_{n}^{5}\right), \end{array}$$

where *A*_2_ = (1 − *g*(0))*c*_2_, *A*_3_ = ((−2 + 4*g*(0) − *g*′(0))*c*_2_^2^ − 2(−1 + *g*(0))*c*_3_), and

(17) A4=((4−13g(0)+7g′(0)−g′′(0)2)c23   +(−7+14g(0)−4g′(0))c2c3−3(−1+g(0))c4).

We now need to vanish *A*_2_ and *A*_3_ not only for making the first two steps optimal but also for simplifying subsequent relations. It is enough to ask the weight function *g* to satisfy conditions *g*(0) = 1 and *g*′(0) = 2. Then

(18) $$\begin{array}{c} e_{z_{n}}=\left(\left(5-\frac{g^{\prime \prime }\left(0\right)}{2}\right)c_{2}^{3}-c_{2}c_{3}\right)e_{n}^{4}+O\left(e_{n}^{5}\right). \end{array}$$

For the third step, we also require

(19) tn=f(zn)f(xn)  =((5−g′′(0)2)c23−c2c3)en3    +((−41+11g′′(0)2)c24      −3(−11+g′′(0))c22c3−2c32−2c2c4)en4  +[(211−73g′′(0)2)c25   +32(−200+27g′′(0))c23c3   +3(23−2g′′(0))c2c32   +12(100−9g′′(0))c22c4−7c3c4]en5+O(en6),

(20) f[zn,yn]=f′(α)[1+c22en2+2c2(−c22+c3)en3     −12c2((−18+g′′(0))c23+14c2c3−6c4)     ×en4]+O(en5),

(21) f[zn,xn,xn] =f′(α)[c2+2c3en+3c4en2−12(c2c3((g′′(0)−10)c22+2c3))en4]+O(en5).

Now let

(22) $$\begin{array}{c} h\left(t_{n}\right)\approx h\left(0\right)+h^{\prime }\left(0\right)t_{n}. \end{array}$$

Taking into account relations (19)–(22) and the third step of (5), we get

(23) en+1=ezn−h(tn)  f(zn)f[zn,yn]+f[zn,xn,xn](ezn−eyn)=B4en4+B5en5+B6en6+B7en7+B8en8+O(en9),

where *B*_4_ = (1/2)(−1 + *h*(0))*c*_2_((−10 + *g*′′(0))*c*_2_^2^ + 2*c*_3_). For the sake of simplicity, we first vanish this coefficient and afterwards the other coefficients will be given in the same strategy. Needless to say, *h*(0) = 1 implies the desired result. Then, imposing this condition, it follows at once that *B*_4_ = *B*_5_ = *B*_6_ = 0 and

(24) B7=−14(c22((−10+g′′(0))c22+2c3)     ×((−10+g′′(0))h′(0)c22+2(−2+h′(0))c3)).

Finally, taking *g*′′(0) = 10 and *h*′(0) = 2, we obtain

(25) $$\begin{array}{c} e_{n+1}=c_{2}^{2}c_{3}\left(28c_{2}^{3}+2c_{2}c_{3}-c_{4}\right)e_{n}^{8}+O{\left(e_{n}\right)}^{9}, \end{array}$$

which shows that under the provided conditions on weight functions *g* and *h* the method (5) has eighth-order convergence and it is optimal. This finishes the proof.

According to the above analysis, we can obtain the following special cases.

**Corollary 2**

If one sets *g*(*s*_*n*_) = (1 + *βs*_*n*_)/(1 + (*β* − 2)*s*_*n*_) = (*f*(*x*_*n*_) + *βf*(*y*_*n*_))/(*f*(*x*_*n*_)+(*β* − 2)*f*(*y*_*n*_)), scheme (14) in [6] is obtained.

Consider

(26) yn=xn−f(xn)f′(xn),zn=yn−f(xn)+βf(yn)f(xn)+(β−2)f(yn)  f(yn)f′(xn), β=−12xn+1=zn−h(tn)f(zn)f[zn,yn]+f[zn,xn,xn](zn−yn),tn=f(zn)f(xn).

**Corollary 3**

If one sets *h*(*t*_*n*_) = (1 + *θt*_*n*_)/(1 + (*θ* − 2)*t*_*n*_) = (*f*(*x*_*n*_) + *θf*(*z*_*n*_))/(*f*(*x*_*n*_)+(*θ* − 2)*f*(*z*_*n*_)), *θ* ∈ *R*, our proposed method becomes scheme (13) in [7].

Consider

(27) yn=xn−f(xn)f′(xn),zn=yn−g(sn)f(yn)f′(xn),  sn=f(yn)f(xn)xn+1=zn−f(xn)+θf(zn)f(xn)+(θ−2)f(zn)×f(zn)f[zn,yn]+f[zn,xn,xn](zn−yn).

In addition to those from Corollaries 2 and 3, some simple but efficient weight functions which satisfy conditions of Theorem 1 are

(28) g1(sn)=2−sn2−5sn=2f(xn)−f(yn)2f(xn)−5f(yn),g2(sn)=11−2sn−sn2=f(xn)2f(xn)2−2f(xn)f(yn)−f(yn)2,g3(sn)=1+2sn+5sn2=f(xn)2+2f(xn)f(yn)+5f(yn)2f(xn)2,h1(tn)=1+θtn1+(θ−2)tn=f(xn)+θf(zn)f(xn)+(θ−2)f(zn), θ∈R,h2(tn)=1+2tn=f(xn)+2f(zn)f(xn).

## 3. Some Concrete Methods

In this section, we put forward some particular three-step methods based on the general class designed in this work.

### 3.1. Methods 1 and 2

Firstly, by combining the methods (26) and (27),

(29) yn=xn−f(xn)f′(xn),zn=yn−f(xn)+βf(yn)f(xn)+(β−2)f(yn)  f(yn)f′(xn), β=−12,xn+1=zn−f(xn)+θf(zn)f(xn)+(θ−2)f(zn)   ×f(zn)f[zn,yn]+f[zn,xn,xn](zn−yn), θ∈R.

Consequently, a special case of (29) appears when *g*_1_(*s*_*n*_) = (2 − *s*_*n*_)/(2 − 5*s*_*n*_) = (2*f*(*x*_*n*_) − *f*(*y*_*n*_))/(2*f*(*x*_*n*_) − 5*f*(*y*_*n*_)) and *h*_1_(*t*_*n*_) = 1/(1 − 2*t*_*n*_) = *f*(*x*_*n*_)/(*f*(*x*_*n*_) − 2*f*(*z*_*n*_)), (*θ* = 0):

(30) yn=xn−f(xn)f′(xn),zn=yn−2f(xn)−f(yn)2f(xn)−5f(yn)  f(yn)f′(xn), β=−12xn+1=zn−f(xn)f(xn)−2f(zn)   ×f(zn)f[zn,yn]+f[zn,xn,xn](zn−yn).

### 3.2. Method 3

Now, let us substitute *g*_1_(*s*_*n*_) = (2 − *s*_*n*_)/(2 − 5*s*_*n*_) = (2*f*(*x*_*n*_) − *f*(*y*_*n*_))/(2*f*(*x*_*n*_) − 5*f*(*y*_*n*_)) and *h*_2_(*t*_*n*_) = 1 + 2*t*_*n*_ = (*f*(*x*_*n*_) + 2*f*(*z*_*n*_))/*f*(*x*_*n*_) into (5). It gives us the following iterative scheme:

(31) yn=xn−f(xn)f′(xn),zn=yn−2f(xn)−f(yn)2f(xn)−5f(yn)  f(yn)f′(xn), β=−12,xn+1=zn−f(xn)+2f(zn)f(xn)   ×f(zn)f[zn,yn]+f[zn,xn,xn](zn−yn).

### 3.3. Method 4

Let us consider *g*_2_(*s*_*n*_) = 1/(1 − *t*_*n*_)^2^ = *f*(*x*_*n*_)^2^/(*f*(*x*_*n*_)^2^ − 2*f*(*x*_*n*_)*f*(*y*_*n*_) − *f*(*y*_*n*_)^2^) and *h*_1_(*t*_*n*_) = 1/(1 − 2*t*_*n*_) = *f*(*x*_*n*_)/(*f*(*x*_*n*_) − 2*f*(*z*_*n*_)), (*θ* = 0). By using them in (5), we have

(32) yn=xn−f(xn)f′(xn),zn=yn−f(xn)2f(xn)2−2f(xn)f(yn)−f(yn)2  f(yn)f′(xn),xn+1=zn−f(xn)f(xn)−2f(zn)   ×f(zn)f[zn,yn]+f[zn,xn,xn](zn−yn).

### 3.4. Method 5

If we consider *g*_2_(*s*_*n*_) = 1/(1 − *t*_*n*_)^2^ = *f*(*x*_*n*_)^2^/(*f*(*x*_*n*_)^2^ − 2*f*(*x*_*n*_)*f*(*y*_*n*_) − *f*(*y*_*n*_)^2^) and *h*_2_(*t*_*n*_) = 1 + 2*t*_*n*_ = (*f*(*x*_*n*_) + 2*f*(*z*_*n*_))/*f*(*x*_*n*_) in (5), we have

(33) yn=xn−f(xn)f′(xn),zn=yn−f(xn)2f(xn)2−2f(xn)f(yn)−f(yn)2  f(yn)f′(xn),xn+1=zn−f(xn)+2f(zn)f(xn)   ×f(zn)f[zn,yn]+f[zn,xn,xn](zn−yn).

### 3.5. Method 6

When *g*_3_(*s*_*n*_) = 1 + 2*s*_*n*_ + 5*s*_*n*_^2^ = (*f*(*x*_*n*_)^2^ + 2*f*(*x*_*n*_)*f*(*y*_*n*_) + 5*f*(*y*_*n*_)^2^)/*f*(*x*_*n*_)^2^ and *h*_1_(*t*_*n*_) = 1/(1 − 2*t*_*n*_) = *f*(*x*_*n*_)/(*f*(*x*_*n*_) − 2*f*(*z*_*n*_)), (*θ* = 0) in (5), we get

(34) yn=xn−f(xn)f′(xn),zn=yn−f(xn)2+2f(xn)f(yn)+5f(yn)2f(xn)2  f(yn)f′(xn),xn+1=zn−f(xn)f(xn)−2f(zn)   ×f(zn)f[zn,yn]+f[zn,xn,xn](zn−yn).

### 3.6. Method 7

Finally, if we consider *g*_3_(*s*_*n*_) = 1 + 2*s*_*n*_ + 5*s*_*n*_^2^ = (*f*(*x*_*n*_)^2^ + 2*f*(*x*_*n*_)*f*(*y*_*n*_) + 5*f*(*y*_*n*_)^2^)/*f*(*x*_*n*_)^2^ and *h*_2_(*t*_*n*_) = 1 + 2*t*_*n*_ = (*f*(*x*_*n*_) + 2*f*(*z*_*n*_))/*f*(*x*_*n*_) in (5), we have

(35) yn=xn−f(xn)f′(xn),zn=yn−f(xn)2+2f(xn)f(yn)+5f(yn)2f(xn)2f(yn)f′(xn),xn+1=zn−f(xn)+2f(zn)f(xn)   ×f(zn)f[zn,yn]+f[zn,xn,xn](zn−yn).

All the methods (29)–(35) require three functional evaluations, namely, *f*(*x*_*n*_), *f*(*y*_*n*_), and *f*(*z*_*n*_), and one of the first derivative, namely, *f*′(*x*_*n*_), per iteration. Therefore, they are optimal in the sense of Kung and Traub's conjecture for *n* = 4 with *p* = 2^3^. Thus, if we assume that all the evaluations have the same cost, then EI = 1.682.

## 4. Numerical Implementation and Comparisons

This section concerns numerical results of the proposed methods (30)–(35). Moreover, they are compared with Kung-Traub's method presented in [8], whose iterative expression is

(36) yn=xn−f(xn)f′(xn),zn=yn−f(xn)(f(xn)−f(yn))2f(yn)f′(xn),xn+1=zn−(1f(xn)−f(zn)(1f[xn,zn]−1f′(xn))      −f(yn)(f(xn)−f(yn))2f′(xn))    ×f2(xn)f(yn)f(yn)−f(zn).

Numerical results have been carried out using Mathematica 8 with 400 digits of precision. In each table, ACOC stands for Approximated Computational Order of Convergence (see [39]), which is given by

(37) $$\begin{array}{c} p\approx \text{ACOC}=\frac{\ln\left(\left|x_{n+1}-x_{n}\right|{\left|x_{n}-x_{n-1}\right|}^{-1}\right)}{\ln\left(\left|x_{n}-x_{n-1}\right|{\left|x_{n-1}-x_{n-2}\right|}^{-1}\right)}. \end{array}$$

Among many test problems, the following four examples are considered:

(38) f1(x)=(x−2)(x6+x3+1)e−x2, α=2, x0=1.8,f2(x)=x2−(1−x)25, α=0.1437392…, x0=2.5,f3(x)=∏k=112(x−k), α=5, x0=5.3,f4(x)=exsin(5x)−2, α=1.3639…, x0=1.2.

From Table 1, it can be seen that all methods work perfectly. Furthermore, we can see that results from methods (34) and (35) are specially good.  Table 2 shows that numerical results are in accordance with their theory well enough. In this example, methods (34) and (35) do not have as good behavior as in Example 1.  Table 3 represents an important case. Although methods (34) and (35) are working very well in Example 1, however, they do not produce convergent iterations here. It should be remarked that these divergent sequences show that some methods work better in some cases, while they may not do it in other ones.

Table 4 shows that all the methods work in concordance with theoretical results.

## 5. Conclusion

A new optimal class of three-step methods without memory has been obtained by generalizing Bi et al. families. This class uses four functional evaluations per iteration and it is optimal in the sense of Kung and Traub's conjecture. Some elements of the family have been presented and they have been tested in order to show its applicability and efficiency, showing that these methods work properly and confirm their theoretical aspects.

## Figures and Tables

**Table 1:** Numerical results with *f*_1_.

Method	|*x*_1_ − *α*|	|*x*_2_ − *α*|	|*x*_3_ − *α*|	ACOC
(30)	0.1378 (−5)	0.7505 (−46)	0.5824 (−368)	8.0000
(31)	0.1487 (−5)	0.1386 (−45)	0.7890 (−366)	8.0000
(32)	0.1304 (−5)	0.6560 (−46)	0.2691 (−368)	8.0000
(33)	0.1417 (−5)	0.1272 (−45)	0.5365 (−366)	8.0000
(34)	0.3811 (−6)	0.2544 (−49)	0.1005 (−394)	8.0000
(35)	0.2083 (−6)	0.2029 (−51)	0.1641 (−411)	8.0000
(36)	0.6210 (−5)	0.1433 (−39)	0.1151 (−316)	8.0000

**Table 2:** Numerical results with *f*_2_.

Method	|*x*_1_ − *α*|	|*x*_2_ − *α*|	|*x*_3_ − *α*|	ACOC
(30)	0.2118 (−3)	0.6382 (−22)	0.4314 (−170)	8.0000
(31)	0.1311 (−3)	0.1375 (−23)	0.2004 (−183)	8.0000
(32)	0.2182 (−3)	0.9901 (−22)	0.1771 (−168)	8.0000
(33)	0.1358 (−3)	0.2225 (−23)	0.1150 (−181)	8.0000
(34)	0.4679 (−3)	0.2262 (−18)	0.7049 (−141)	7.9999
(35)	0.3139 (−3)	0.9409 (−20)	0.6319 (−152)	7.9999
(36)	0.2165 (−3)	0.1415 (−23)	0.4944 (−185)	7.9990

**Table 3:** Numerical results with *f*_3_.

Method	|*x*_1_ − *α*|	|*x*_2_ − *α*|	|*x*_3_ − *α*|	ACOC
(30)	0.2222 (−2)	0.5371 (−22)	0.5914 (−179)	8.0012
(31)	0.2197 (−2)	0.4897 (−22)	0.2824 (−179)	8.0012
(32)	0.8787 (−1)	0.2317 (−9)	0.3280 (−78)	8.0254
(33)	0.5637 (−1)	0.1005 (−10)	0.4105 (−89)	8.0407
(34)	0.1791 (1)	0.2000 (1)	0.2000 (1)	—
(35)	0.4703 (1)	0.4584 (27)	0.9577 (26)	—
(36)	0.2210 (−1)	0.5727 (−13)	0.1870 (−105)	7.9823

**Table 4:** Numerical results with *f*_4_.

Method	|*x*_1_ − *α*|	|*x*_2_ − *α*|	|*x*_3_ − *α*|	ACOC
(30)	0.2119 (−4)	0.1307 (−36)	0.2749 (−294)	8.0000
(31)	0.1731 (−4)	0.2597 (−37)	0.6668 (−300)	8.0000
(32)	0.2333 (−4)	0.2789 (−36)	0.1166 (−291)	8.0000
(33)	0.1973 (−4)	0.7310 (−37)	0.2599 (−296)	8.0000
(34)	0.1769 (−4)	0.2191 (−37)	0.1212 (−300)	8.0000
(35)	0.1767 (−4)	0.2177 (−37)	0.1153 (−300)	8.0000
(36)	0.2074 (−4)	0.2571 (−36)	0.1433 (−291)	8.0000
